# Resolving Shifting Patterns of Muscle Energy Use in Swimming Fish

**DOI:** 10.1371/journal.pone.0106030

**Published:** 2014-08-28

**Authors:** Shannon P. Gerry, David J. Ellerby

**Affiliations:** 1 Biology Department, Fairfield University, Fairfield, Connecticut, United States of America; 2 Department of Biological Sciences, Wellesley College, Wellesley, Massachusetts, United States of America; University of Utah, United States of America

## Abstract

Muscle metabolism dominates the energy costs of locomotion. Although in vivo measures of muscle strain, activity and force can indicate mechanical function, similar muscle-level measures of energy use are challenging to obtain. Without this information locomotor systems are essentially a black box in terms of the distribution of metabolic energy. Although in situ measurements of muscle metabolism are not practical in multiple muscles, the rate of blood flow to skeletal muscle tissue can be used as a proxy for aerobic metabolism, allowing the cost of particular muscle functions to be estimated. Axial, undulatory swimming is one of the most common modes of vertebrate locomotion. In fish, segmented myotomal muscles are the primary power source, driving undulations of the body axis that transfer momentum to the water. Multiple fins and the associated fin muscles also contribute to thrust production, and stabilization and control of the swimming trajectory. We have used blood flow tracers in swimming rainbow trout (*Oncorhynchus mykiss*) to estimate the regional distribution of energy use across the myotomal and fin muscle groups to reveal the functional distribution of metabolic energy use within a swimming animal for the first time. Energy use by the myotomal muscle increased with speed to meet thrust requirements, particularly in posterior myotomes where muscle power outputs are greatest. At low speeds, there was high fin muscle energy use, consistent with active stability control. As speed increased, and fins were adducted, overall fin muscle energy use declined, except in the caudal fin muscles where active fin stiffening is required to maintain power transfer to the wake. The present data were obtained under steady-state conditions which rarely apply in natural, physical environments. This approach also has potential to reveal the mechanical factors that underlie changes in locomotor cost associated with movement through unsteady flow regimes.

## Introduction

Locomotion is energetically costly [1], [2], and the vast majority of this energy is directed to the skeletal musculature [3], [4], [5]. Skeletal muscles perform many mechanical roles; acting as a power source, accelerating body or limb segments, or transferring momentum to a fluid [6], [7], [8]. In some circumstances they may also act like a strut, to transfer force or power [9], [10], or a brake, absorbing energy to exert control and maintain stability [11]. The anatomical complexity of vertebrate musculoskeletal systems combined with this functional diversity presents a major barrier to understanding how multiple muscles interact to power and control movement.

A first, basic step in understanding the importance of particular mechanical functions within a locomotor system is to determine how much energy is directed to muscle groups associated with those functions. Such information has been lacking, as determining the distribution of energy expenditure throughout the skeletal musculature presents major methodological challenges. Direct measures of energy expenditure by muscles or muscle groups typically require concurrent measurements of blood flow rates and oxygen levels, deriving rates of muscle oxygen consumption via the Fick principle [12], [13], [14]. Anatomical constraints limit the application of this approach, and energy expenditure cannot be resolved simultaneously in multiple muscles. Therefore, in the absence of systemic data indicating muscle energy use, studies of locomotor energetics and muscle mechanical function have largely been confined to either the whole organism or single muscle level. Measurements of total energy expenditure have been obtained from many animals during multiple modes of locomotion, yielding valuable information such as the total cost of transport [1], [15], [16]. Muscle level measurements of activity, strain and force production have revealed the mechanical roles of many specific muscles in locomotion [17], [18], [19], [20]. Without a link between mechanical function and energy expenditure at the muscle level however, the relative importance of a particular muscle’s mechanical contribution to locomotion is impossible to assess [21], [22].

The rate of blood flow to a muscle provides a means of resolving energy use at the muscle level, enabling the link between energy expenditure and mechanical function to be established. Concurrent measurements of blood flow rate and aerobic metabolism in skeletal muscle have established that blood flow rate is proportional to the muscle’s rate of oxygen consumption [5], [13], [23], [24], [25], [26]. Blood flow can therefore be used as a proxy measure of energy expenditure during aerobically supported exercise [21], [27], [28]. This technique has so far only been applied to investigate the distribution of energy use within the leg musculature of birds during terrestrial locomotion [5], [28], [29], [30], but has the potential to reveal the distribution of energy expenditure within the skeletal musculature during other modes of vertebrate locomotion.

The skeletal musculature of swimming fish performs a number of mechanical functions. In undulatory swimmers the segmented, myotomal muscle provides power for thrust generation [10]. This muscle type is not a simple motor. The segmented architecture of the myotomal musculature is highly complex, and shows regional differentiation in function between muscle fiber types, and with location along the body axis [6], [10], [31], [32]. Analyses of fin muscle activity and the contributions of fins to thrust and stability have highlighted the importance of this part of the musculature in both steady swimming, and unsteady maneuvers [33], [34], [35], [36], [37], [38], [39]. The techniques applied in these studies, revealing kinematics, muscle strain, activity and contractile properties, give valuable information regarding muscle function. This information is however localized to the specific site from which the data were obtained. Given the complexity of the myotomal architecture, and the large number of fins and associated muscles contributing to swimming thrust and control, an integrated overview of muscle function during swimming is unavailable. Our goal was to use regional variation in skeletal muscle blood flow as a tool to determine the relative energetic costs of myotomal and fin muscle function during steady, undulatory swimming in trout; allowing costs to be ascribed to particular muscle functions during swimming for the first time.

It is likely that these relative costs shift with swimming speed. The fins are important for stabilizing fish trajectory during swimming. This can be achieved by trimming, extending the fins to produce lift-based stabilizing forces, or by powered fin movements [40], [41]. As lift-forces are approximately proportional to the square of flow velocity, the requirement for stabilization by fin movements, and therefore the greatest relative cost for fin control should occur at low speeds where lift-based trimming forces are minimal. Regional shifts in energy expenditure within the fin musculature are also likely. Median and paired fins deployed to maintain stability at low speeds may be retracted against the body at higher speeds [33], [34], [36], while recruitment of the intrinsic muscles of the caudal fin increases with speed [35]. This leads us to hypothesize that as swimming speed increases energy use by fin muscles will shift from the paired fins, dorsal and anal fins that actively stabilize the fish at low speeds, to the caudal fin, with its primary role in mechanical power transfer to the water.

Swimming thrust requirements increase in approximate proportion to the cube of swimming velocity. The myotomal muscle is the primary mechanical power source in undulatory swimmers. Therefore, given the potential decline in stability costs with speed, energy expenditure by the myotomal muscle should represent a greater proportion of total swimming costs as speed increases. Muscle activity data suggest that the myotomal muscle also contributes to stability in ways that change with speed. For example, at low swimming speeds, the anterior myotomal slow muscle shows variable patterns of activity and strain that produce low power outputs, while the posterior myotomes function more consistently as a power source [42]. As speed increases anterior muscle function is less variable, and power output increases. This is compatible with a functional shift in the anterior myotomal muscle from stabilization at low speeds, where corrections are made to posture and heading, to power production at high speeds. Given the potential low speed stabilization role of the anterior myotomal musculature, and that trout slow muscle mass and mass specific power output are greatest in the posterior myotomes [11], [43], we hypothesize that overall myotomal energy use will increase with speed, and that the support of power production in posterior muscle will represent an increasing proportion of the total cost.

Blood flow measurements also have the potential to resolve ambiguity regarding functional and regional divisions in activity within the myotomal musculature. In the myotomes of many fish, there is a clear anatomical and functional division between red, aerobic and white, glycolytic muscle fibers [44], [45]. These are physiologically suited to different roles, red fibers for powering slower, sustained swimming, and white fibers for powering brief, unsustained movements [46], [47]. In salmonids the anatomical and functional subdivisions are less clear cut. The bulk of the myotomal muscle, consisting of white fibers in most other fish, is of a mixed, ‘mosaic’ type, with small-diameter fibers superficially resembling red muscle interspersed within the white fibers [48], and forming a proportion of the total myotomal muscle mass approximately equal to that of the superficial red muscle [49]. These interspersed fibers may however be physiologically distinct from red muscle [50]. Their function and the contribution of the mosaic muscle as a whole to sustained swimming activity is unclear. Indirect evidence, such as increased mosaic muscle capillary density and mosaic muscle hypertrophy in response to sustained swimming, suggest recruitment of mosaic muscle at relatively low, aerobically supported swimming speeds [51], [52], [53]. Direct measurements of mosaic muscle activity are less consistent. In some cases activity is detected at aerobic speeds [54], including low levels of activity attributed to the red mosaic fibers [55], while other studies have detected no activity until speeds that likely exceed the aerobic capacity of the fish [56]. These contrasting results may be due to the localized nature of EMG recordings, and differences in electrode placement and geometry between studies. Blood flow measurements obtained throughout the entire myotomal musculature should provide a more complete picture of myotomal activity and energy use.

In order to examine speed-related shifts in muscle blood flow and energy distribution, rainbow trout were swum at a range of aerobically supported speeds in a recirculating flume. Total aerobic energy expenditure was estimated by indirect calorimetry using closed system respirometry. Blood flow distributions were measured using fluorescently labeled microspheres introduced into the systemic circulation via a dorsal aortic cannula. These lodged in systemic capillaries in trace amounts proportional to the blood flow rate. Microspheres were extracted from target tissues post mortem by chemical digestion and sedimentation, and their numbers determined by spectrofluorimetry based on the concentration of fluorescent dye extracted from the recovered spheres with a solvent. Spheres with different excitation/emission fluorescent spectra allowed for distributions to be measured within the same individual at several different speeds. The proportionality between skeletal muscle blood flow rate and muscle aerobic metabolism was used to estimate speed-related changes in the proportion of the total metabolic cost of swimming directed to particular muscles and muscle groups, revealing the shifting patterns of energy use within a swimming fish for the first time.

## Materials and Methods

### Study Animals

Rainbow trout, *Oncorhyncus mykiss* (N = 8, total length, *L* = 0.296±0.018 m; mass = 0.311±0.042 kg, mean ± sem), were obtained from the Sandwich Hatchery (Sandwich, MA, USA). Trout were housed in 500 l collapsible koi tanks at a temperature of 15°C on a 12∶12 light:dark cycle. They were fed commercial trout pellets three times per week. All procedures were approved by the Institutional Animal Care and Use Committee at Wellesley College, and were carried out in accordance with the recommendations in the Guide for the Care and Use of Laboratory Animals of the National Institutes of Health.

Two sets of experiments were carried out in sequence. In the first, measurements of oxygen consumption during incremental speed changes were used to establish the overall aerobic cost of swimming and the limits of aerobic performance for each individual. In the second, regional blood flow measurements with injected flow tracers were carried out at rest, and at three swimming speeds defined as proportions of the maximal aerobically supported speed determined for that individual. A minimum of 4 days recovery was allowed between experiments.

### Oxygen Consumption

Swimming experiments were performed in a recirculating flume (Loligo Systems, Tjele, Denmark), sealable for measurements of oxygen concentration at 15°C. The flume had a working section 20 cm wide×20 cm deep×70 cm long, maximum flow rate of 200 cm s^−1^, 88.6 L volume, and was calibrated for flow velocity. Fish were allowed to acclimate in the flume overnight before all experiments and fasted for 48·h, to avoid postprandial rises in the rate of oxygen consumption 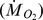.

Progressive increments in swimming speed, imposed in the flume, were used to establish the maximum sustainable swimming speed for individual fish (*U_max_*). Starting at 8.4·cm·s^–1^, approximately 0.3 *L* s^−1^, speed was increased in 4·cm·s^–1^ increments and maintained at a given level for 20·min between increments. Speed increases continued until the fish could no longer maintain position in the flume. This coincided with the onset of burst and coast swimming behavior and high amplitude lateral body undulation, indicative of the recruitment of anaerobic muscle. This approximately defines the upper limit of the speed range powered aerobically by slow muscle. The time maintained at the final speed interval (*T*, in min) was recorded. *U_max_* (in cm·s^–1^) = *U_fin_* +4(*T*/*T_int_*), where *U_fin_* (in cm·s^–1^) was the speed of the final interval at which steady swimming could be maintained and *T_int_* was the time interval between speed increments. This test was not equivalent to a critical swimming speed (*U_crit_*) test, as these typically involve exercising the fish until complete exhaustion, indicated by an inability to move from the mesh at the rear of the flume working section [57]. Our aim was to determine maximal aerobically supported performance in a similar manner to Claireaux et al. [58]. Speeds approaching *U_crit_* involve the recruitment of anaerobic muscle [59], [60], elevating performance above sustainable, aerobic levels. A standard *U_crit_* protocol was therefore not compatible with our experimental goals.


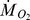
 was calculated from the rate of decline of oxygen concentration in the sealed flume during the speed increments used to establish *U_max_*. Initial oxygen concentration measurements were taken in an empty flume to determine the rate of oxygen consumption by the oxygen electrode and microorganisms in the flume. These readings were repeated after obtaining data for each fish and were subsequently subtracted from all fish 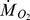 values. Flume volume was corrected for the volume of water displaced by the fish (calculated from body mass, assuming an average density equal to water). Mass-specific 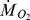 = *R*[(*V_flume_*–*V_fish_*)/*M*]·mg O_2_·kg^–1^·h^–1^, where *R* is the measured rate of oxygen decline in the sealed flume in mg O_2_·l^–1^·h^–1^, *V_flume_* is the flume volume in liters, *V_fish_* is the volume displaced by the fish, and *M* is the body mass of the fish in kg.

Cannulation had no detectable effect on 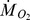 or *U_max_*. Even with double PE50 aortic cannulations in rainbow trout of this size, there are no detectable effects on total swimming cost or maximal performance [61], although net swimming costs, minus resting metabolism, are elevated at speeds approaching *U_crit_*.

### Microsphere Injections

The microspheres (Fluogen, Invitrogen, Thermo Fisher, USA) were supplied suspended in sterile physiological saline containing 0.05% Tween-80 and 0.01% thimerosal at a density of 3 million spheres per ml. For each batch of spheres of each color, we determined a standard curve of fluorescence following excitation at the recommended wavelength for a range of concentrations based on the dye extracted with cellosolve acetate from a known mass of the well-mixed suspension of spheres. Microsphere suspensions were vortexed for 45 s prior to being drawn up into the injection syringe to ensure even suspension of the spheres. Syringes were weighed to the nearest milligram before and after filling to give a precise measure of the amount of microsphere solution in the syringe. The amount of spheres actually injected was determined from the amount in the syringe minus the residual spheres remaining in the syringe tip and the injection port. Approximately 1×10^5^ spheres were injected at each level of exercise.

Microspheres were injected via a heparin coated PE50 cannula inserted into the dorsal aorta. Cannulations were carried out under MS222 anesthesia following standard techniques [62], [63]. Briefly, a sharpened wire trochar was used to insert the cannula through the tissue in the midline of the roof of the mouth adjacent to the 2^nd^ gill arch and into the dorsal aorta. The trochar was withdrawn and the cannula filled with heparinized saline and plugged. The cannula was anchored to the roof of the mouth by sutures, and exited the mouth via a hole made from a naris into the mouth. The hole was lined with a grommet made from PE160 tubing, heat flared at the internal end to prevent it pulling out through the hole. The PE50 cannula was routed back from the exit point and secondarily sutured to the skin anterior to the dorsal fin. Fish recovered for up to 12 hours in the swimming flume before microsphere injections.

The tip of the cannula was located anterior to the coeliaco-mesenteric artery that supplies the liver, digestive system, spleen and swim bladder and the subclavian arteries that serve the pectoral girdle, but posterior to the branches that supply the eyes, brain, coronary circulation and musculature of the head. The flow distributions determined from our microsphere injections therefore do not account for this portion of total cardiac output. At the speeds chosen, the trout primarily relied on ram ventilation of the gills, although some ventilatory movements may have occurred. Ram ventilation costs are largely borne by the swimming musculature providing thrust to overcome the associated drag, rather than the musculature associated with the jaw and operculum whose blood flow rates and energy expenditures should therefore be low [64]. At rest the salmonid coronary circulation receives approximately 1% of cardiac output [65], [66] and has a maximum 2.5 fold scope for increased coronary flow in rainbow trout [67]. In energetic terms the costs of oxygen delivery, the primary energetic demand placed on the part of the circulation not reached by the microspheres is between 5 and 0.5% of the total swimming energy cost across the ram ventilation speed range [68]. Given the relatively low mass (<0.9% of total body mass for the musculature of the jaw and operculum) and energy expenditure of the systemic tissues not receiving microspheres, the effects on estimates of blood flow distribution and the associated muscle energy expenditure should be small even with some active respiratory movements.

Trout were injected with four colors of 15 µm diameter microspheres (FluoSpheres, Invitrogen), one injection color for each speed. Microspheres were checked for dimensional accuracy and uniformity under a light microscope. Their diameter and density (1.05 g ml^−1^) approximates that of trout erythrocytes and was chosen to exceed that of trout capillaries (3 µm, [69]) and has previously been shown to be captured by the systemic capillaries of trout, with minimal return to the gills [55], [70], [71]. In the present experiments this percentage was less than 1% of the total injected. Smaller 10 µm diameter microspheres may not lodge in the systemic vessels, resulting in relatively high capture rate in the gills (>5%, [72]). Further increasing microsphere diameter, although it may ensure greater lodging in systemic vessels, is undesirable as they will come to rest in vessels increasingly further removed from the actual site of exchange in the capillaries, and will occlude flow to a larger number of downstream arterioles and capillaries.

Prior to the injection series, the cannula plug was removed and replaced with an injection port (SIP22/4, Instech Solomon, Plymouth Meeting, PA, USA). The trout were injected while at rest (flume flow speed 0.2 *L* s^−1^ at which the trout rested at the bottom, rear of the working section) and at swimming speeds of 0.5, 0.75 and 0.95 *U_max_*. This speed range was chosen for a number of reasons. In order to link blood flow rate to oxygen delivery, the oxygen carrying capacity of the blood and the oxygen extraction efficiency need to remain relatively constant. In salmonids, arterial blood remains oxygen saturated across the entire speed range up to *U_crit_* [73], [74], [75], [76]. In trout, venous oxygen tensions are reduced at intense levels of swimming effort approaching *U_crit_* [73], [77], [78] but do not change detectably at more moderate, aerobically supported speeds up to 80% of *U_crit_* at the temperature used in the present study [78]. This suggests that both the oxygen carrying capacity of the blood and tissue extraction remain relatively constant across our chosen level of effort. Changes in oxygen demand by the tissues are therefore largely met by increased blood flow [79], [80].

The order of the speed conditions was selected randomly for each fish. Trout swam for a minimum of 10 minutes at the required speed. After a stepwise speed change, cardiac output and systemic blood pressure adjust and stabilize at the new level within approximately 3 minutes [66]. Preliminary validation experiments indicated that 4 successive injections of this number of spheres could be given without causing any significant effect on swimming performance. Additional validation trials indicated that blood flow measurements were reproducible when successive injections were done at the same swimming speed. A lack of effect on cardiovascular function was confirmed by monitoring heart rate and cardiac output before and during the trial involving injection of microspheres in a subset of fish with a cuff-type Doppler flow probe fitted around the ventral aorta [81]. After completion of each microsphere injection the cannula was flushed with phosphate-buffered saline and the speed maintained for a minimum of 2 minutes. Between injection speeds the flume speed was reduced to 0.2 *L* s^−1^ for 15 minutes, sufficient time for heart rate and cardiac output to return to resting levels.

It has been suggested that microsphere measurements may systematically underestimate blood flow to the gastrointestinal tract [74]. Microsphere measurements are available from several studies. In anesthetized albacore tuna (*Thunnus alalunga*), approximately 18% of cardiac output was directed to the gastrointestinal tract [82]. For resting rainbow trout, values range from approximately 16% (present study; [83]) to over 30% ([70], based on reported mass specific flows to liver and intestines and estimated liver and intestinal masses of 1.0 and 2.0% of body mass calculated from tissue masses obtained in the present study). Cameron [84] does not report blood flow to the stomach or intestines, but detected relative flows of approximately 13% to the liver alone in resting arctic grayling (*Thymallus arcticus*). The lowest values were found in the channel catfish (*Ictalurus punctatus*) and the largescale sucker (*Catostomus macrocheilus*) where resting gastrointestinal flows were only 3% of cardiac output [71], [85]. In comparison, direct flow cuff measurements of percent of cardiac output directed through the coeliaco-mesenteric artery at rest were 16 to 19% in white sturgeon (*Acipenser transmontanus*, [86]), 40% for cod (*Gadus morhua*, [81]), and 36% for Chinook salmon (*Oncorhynchus tshawytscha*, [74]), although absolute cardiac output was not measured simultaneously in the latter two studies.

Although the lowest estimates of gastrointestinal blood flows come from microsphere studies, the ranges of values obtained from both microsphere and flow cuff measurements of blood flow do not suggest a systematic difference. There are however methodological reasons that could lead microsphere measurements to underestimate, or produce more variable measures of gastrointestinal flows. Resting gut blood flow rates can drop abruptly in a resting fish, for example during a bout of movement or struggling [71], [87]. Transient decreases in flow are obvious in flow cuff data and can be excluded from analyses. Coincidence of a microsphere injection with such a transient change may be less clear. Also, flow cuff data are typically averaged over longer time periods than those taken for the injection and circulation of microspheres, minimizing the potential effect of transient flow changes. Of greater concern is the potential proximity of the branch point of the coeliaco-mesenteric artery to the tip of the injection cannula [86]. This may lead to incomplete mixing of microspheres into the systemic flow before the branch point, or in some cases detection of no gastrointestinal flow if the injection point is downstream from the branch. This may be a factor in variable data sets where gastrointestinal flows are high in some individuals but effectively zero in others [85], or where there is a mismatch between cuff measured and microsphere based flow rates [86]. Post-mortem dissection to confirm a sufficiently anterior cannula placement and/or exclusion of data with zero gastrointestinal flows [83] can minimize the potential for error. The general requirements for accurate microsphere measurements of blood flow are discussed further by Marsh & Ellerby [21].

### Microsphere Recovery

Following completion of the microsphere injections the trout were euthanized by overdose of MS222. Tissue samples and organs were placed in labeled centrifuge tubes for processing. The myotomal musculature was divided into 10 transverse segments of equal width. Slow myotomal muscle was separated from the underlying mosaic muscle in each segment. The muscles associated with each fin were also removed. In some cases multiple small muscles were combined into functional groups e.g. pectoral fin abductors. All internal organs were also dissected for processing. Large tissue samples were processed in their entirety rather than sub-sampled to avoid errors in blood flow estimation due to small-scale heterogeneity in blood flow rate (reviewed in [88]). The unprocessed tissues primarily consisted of bone and cartilage, which were assumed to have low blood flow rates, and the skin, which also receives relatively little blood flow. Tissues and organs with a mass of less than 2 g were placed in 15 ml polypropylene centrifuge tubes (Falcon Blue Max 2096, Becton Dickinson, USA). Those with a mass of 1 - 9 g were placed in 50 ml centrifuge tubes (Falcon Blue Max 2070, Becton Dickinson, USA). Tissues/organs larger than 9 g were subdivided into multiple tubes to allow maintenance of a sufficiently high ratio of liquid to tissue during subsequent processing.

The tissues were digested and the microspheres recovered by centrifugation following a protocol similar to Marsh et al. [28]. Prior to any further processing a known amount of Navy microspheres was placed in each tube. Because Navy was a color not injected into the animals, it acted as a process control to allow correction for loss of microspheres during the tissue processing. Typically 70 to 90% of the microspheres were successfully recovered. Digestion of the tissues was initiated by adding 1 M KOH to the tubes (approximately 15∶1 ratio, KOH: tissue by volume) and placing them in an oven at 60°C. The samples were digested for 24–48 h with periodic vortex mixing. After digestion was complete the tubes were centrifuged at 1500 g for 15 minutes. A vacuum aspirator was used to remove the supernatant to a level above the pellet without any resuspension. The pellet was resuspended by sonicating in dionized water at 50°C. Subsequent cycles of refilling, sonication, and centrifugation were carried out using, in sequence: dionized water at 50°C; 15% Triton X-100; acidified ethanol (100% ethanol containing 0.5 ml concentrated HCl per liter); and 100% ethanol. After the final centrifugation and aspiration step, the remaining ethanol was allowed to evaporate overnight at room temperature. At this stage virtually all the tissue residue had been removed leaving a clean pellet of microspheres. Cellosolve acetate was added to the dry microsphere pellets, and the spheres resuspended and dissolved by vortex mixing to extract the fluorescent dyes. After centrifugation, the fluorescence spectra of the dye mixtures in cellosolve acetate were measured using a fluorescence spectrometer (LS55, Perkin Elmer, Santa Clara, CA, USA). Samples were scanned across a range of wavelengths from 350 to 725 nm. The numbers of microspheres of the four experimental colors and the Navy process control were calculated from the fluorescence standard curves obtained for each color. The amount of cellosolve acetate in each sample was adjusted to keep peak fluorescence within the measurement range of the spectrometer.

### Statistical Analyses

All statistical analyses were carried out using PASW (Version 18, IBM SPSS, NY, USA). General linear models (GLM) with speed and tissue type as fixed factors, and relative blood flow rates and estimated muscle energy expenditure as dependent variables were used to test for speed-tissue type interaction effects. An individual identifier for each fish was initially included as a random factor in each model. Where no overall differences were detected based on the individual identifier, and no interaction effects with the individual identifier were detected, this factor was removed from the statistical models. *η* ^2^ is reported with GLM outputs as an indicator of effect size. Planned contrasts were used to test for changes in blood flow or energy expenditure in a given muscle or muscle grouping with respect to changes in swimming speed, or to determine whether measured blood flows differed from predictions based on proportional muscle mass.

## Results

### Aerobic cost of swimming

**Figure 1:**
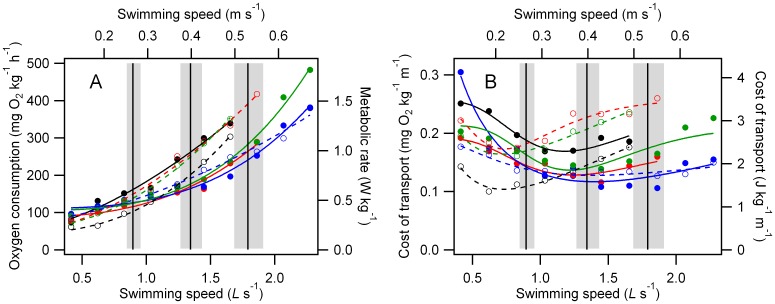
A. Rate of rainbow trout oxygen consumption during steady swimming. B. Total cost of transport during steady swimming. Vertical black lines and gray boxes show the mean ± sem of the speeds used for blood flow measurements (0.50, 0.75, and 0.95 *U_max_* for each fish). Data from each individual are represented by a separate color/symbol type. Data in A are fitted with power functions, and B with log-normal functions. Dashed lines are associated with open symbols of the same color, and unbroken lines with closed symbols. Absolute swimming speeds shown on the top axis are an approximation based on mean fish length. Conversion of oxygen consumption rates to energy units was made with an oxycaloric value of 13.54 J mg^−1^ O_2_ [89]. N = 8.

Swimming 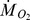 had a curvilinear relationship to swimming speed (Fig. 1A). The mean resting 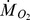 estimated by extrapolation of the 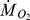-speed relationship to zero speed was 72.3±9.6 mg O_2_ kg^−1^ h^−1^ (± sem). Mean *U_max_* was 1.79±0.12 *L*s^−1^ (± sem). The minimum COT was 0.14±0.01 mg O^2^ kg^−1^ m^−1^, occurring at a minimum cost speed of 1.08±0.11 *L*s^−1^ (± sem). For most fish however, the relatively flat cost-speed relationship close to the minimum cost speed meant that a close to minimal COT was achieved at a range of intermediate speeds (Fig. 1B). COT typically increased as speed approached *U_max_*. A similar increase at low speeds may reflect the increased costs of active stabilization and the associated induced drag, but is also an inevitable consequence of their being a finite cost for swimming at speeds approaching zero, creating an asymptotic relationship.

### Regional blood flow distributions

**Figure 2:**
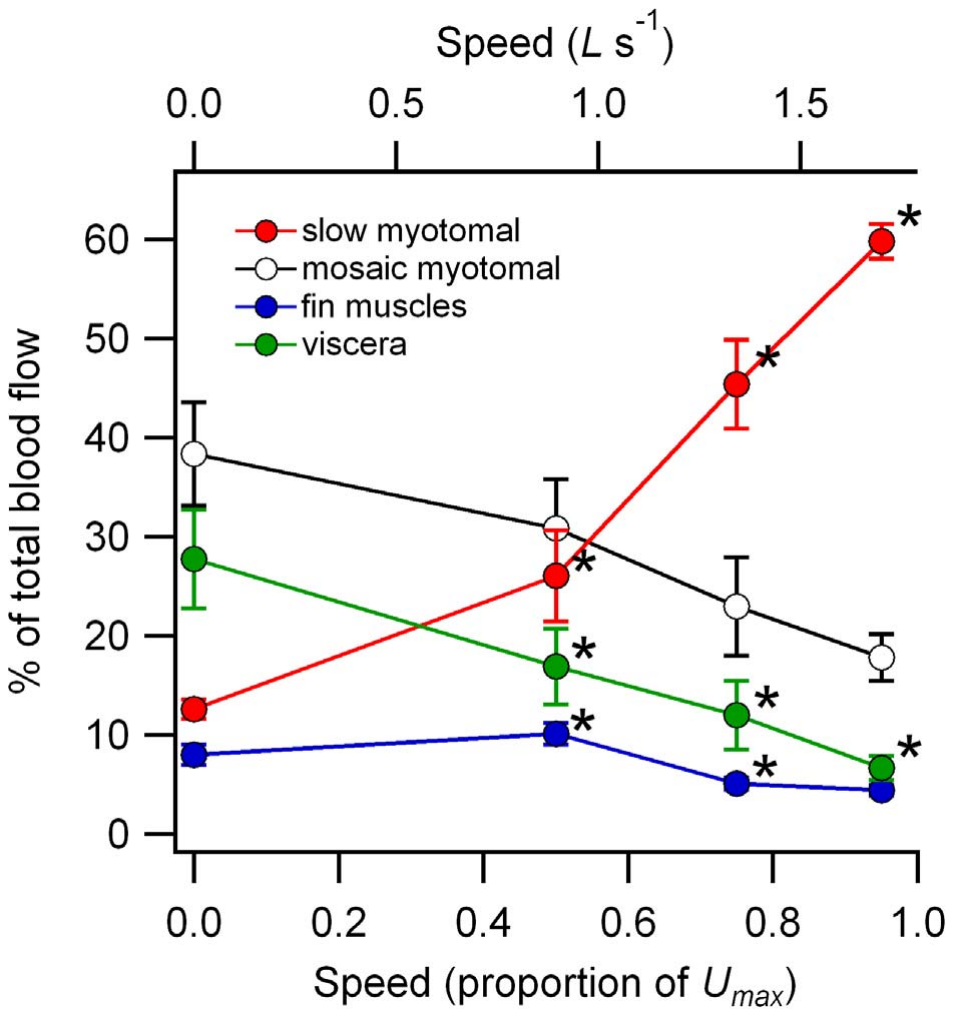
Percentage of total blood flow directed to the slow and mosaic myotomal musculature, fin musculature and viscera. Data are shown as mean ± sem (N = 6 to 8). * designates a significant change relative to the adjacent lower speed condition detected using a planned contrasts procedure (p<0.05).

There was a significant speed-tissue type interaction for proportional blood flow distributions (GLM, F_9,108_ = 16.1, p<0.001, *η* ^2^ = 0.57), supporting the visual impression of differing patterns of distribution in relation to speed for each tissue category (Fig. 2). There was a progressive increase in the percentage of blood flow directed to the slow myotomal muscle with increasing speed, while visceral percentages decreased with speed. The percentage of blood flow to the fin muscles was greatest at the lowest swimming speed. No speed-to-speed changes in percent blood flow to the mosaic myotomal muscle were detected, although a one-way GLM analysis restricted to the mosaic muscle data detected a moderate speed effect in relation to the percentage of blood flow distributed to that muscle type (GLM, F_3,27_ = 3.7, p = 0.025, *η* ^2^ = 0.29), consistent with the overall pattern of decrease relative to rest with increasing speed.

**Figure 3:**
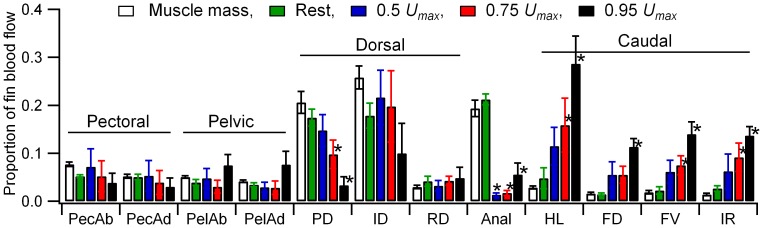
Proportional distribution of blood flow within the fin musculature. Flows are expressed as a proportion of the total blood flow directed to the fin musculature. Muscle mass is expressed as a proportion of the total muscle mass. * denotes a significant difference between the blood flow and muscle mass proportions based on a planned contrasts procedure using muscle mass as the comparison group (p<0.05). Data are shown as mean ± sem (N = 6 to 8). Muscles are as follows: PecAb, pectoral fin abductors; PecAd, pectoral fin adductors; PelAb, pelvic fin abductors; PelAd, pelvic fin abductors; PD, protractor dorsalis; ID, inclinator dorsalis; RD, retractor dorsalis; Anal, anal fin musculature; HL, hypochordal longitudinalis;FD, flexor caudalis dorsalis; FV, flexor caudalis ventralis; IR, interfilamenti caudalis.

We detected a significant interaction effect between fin muscle type and the mass/speed condition (GLM, F_44,396_ = 2.7, p<0.001, *η* ^2^ = 0.23) indicating differences in the pattern of flow distribution in relation to muscle mass and swimming speed between fin muscle types. At rest, blood flow to the fin muscles was in proportion to their mass. During steady swimming the caudal fin muscles received disproportionately high blood flows in relation to their mass, and flows to the anal fin muscles, and protractor dorsalis of the dorsal fin were disproportionately low (Fig. 3). No speed-related changes in relative blood flow distribution compared to muscle mass were detected in the muscles of pectoral and pelvic girdles, or the inclinator dorsalis and retractor dorsalis of the dorsal fin.

### Partitioning total swimming costs

**Figure 4:**
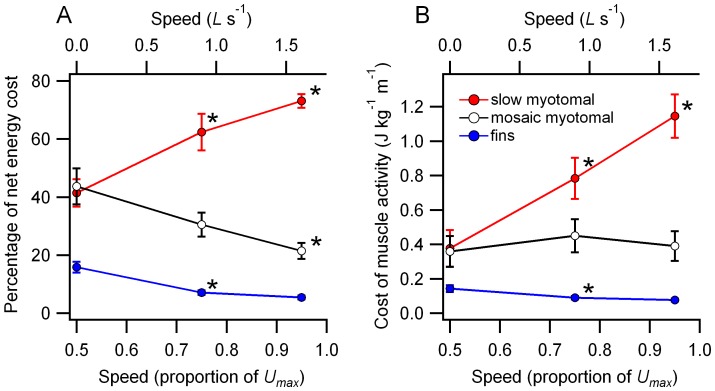
A. Estimated percentage of swimming energy costs directed to the myotomal and fin musculature during steady swimming. B. Estimated net cost of transport associated with activity in the myotomal and fin musculature. Data are shown as mean ± sem (N = 6 to 8). * designates a significant change relative to the adjacent lower speed condition.

The net cost of swimming was estimated by subtracting resting 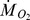 from the total swimming 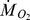 as a means of removing energy costs not directly associated with swimming (see Discussion). For a given muscle category (fins, slow and mosaic myotomal) the amount of swimming energy directed to that category for each swimming speed was estimated by multiplying the net energy cost at that speed by the proportion of blood flow directed to that category. A small proportion of cardiac output is unaccounted for due to the injection site of our microsphere flow tracers, but this is estimated to be less than 5% of total cardiac output (see Materials & Methods). This allowed the relative and absolute energy costs of muscle activity within each category to be estimated using an oxycaloric value of 13.54 J mg^−1^ O_2_ [89] (Fig. 4). Costs are expressed as a cost of transport for the total mass of the fish in J kg^−1^ m^−1^. There was a significant speed-muscle type interaction for both percent and absolute muscle activity costs (GLM, F_4,60_ = 11.04, p<0.001, *η* ^2^ = 0.42, percent cost; F_4,60_ = 4.48, p<0.01, *η* ^2^ = 0.23, absolute cost). The estimated relative and absolute energy costs of slow muscle activity increased significantly with speed (Fig. 4). Estimated costs of fin muscle activity fell from 0.5 to 0.75 *U_max_*, with no further detectable change at the highest swimming speed. Estimates of absolute energy use by the mosaic myotomal muscle did not change detectably with speed (Fig. 4B), this was associated with decreased relative energy use by this muscle type (Fig. 4A).

**Figure 5:**
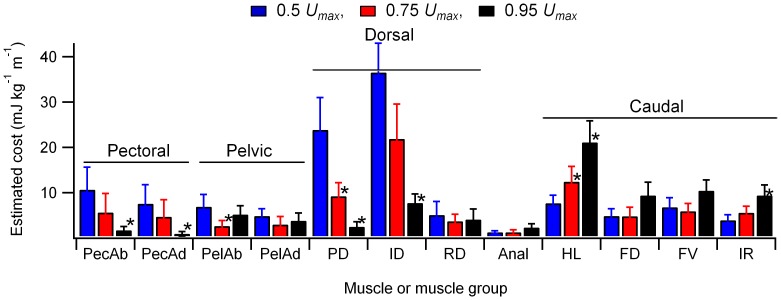
Estimated energy costs of activity within the fin musculature. Energy estimates were obtained by multiplying the total estimated energy directed to the fin muscles, expressed as a net cost of transport, by the proportion of fin muscle blood flow received by each muscle or muscle group. Data are shown as mean ± sem (N = 6 to 8). * designates a significant change relative to the adjacent lower speed condition. Muscles are as follows: PecAb, pectoral fin abductors; PecAd, pectoral fin adductors; PelAb, pelvic fin abductors; PelAd, pelvic fin abductors; PD, protractor dorsalis; ID, inclinator dorsalis; RD, retractor dorsalis; Anal, anal fin musculature; HL, hypochordal longitudinalis;FD, flexor caudalis dorsalis; FV, flexor caudalis ventralis; IR, interfilamenti caudalis.

To provide a more detailed regional estimate of energy use within the fin musculature, the total estimated energy directed to the fin musculature was multiplied by proportion of the total fin muscle blood flow received by a given muscle or muscle group (Fig. 5). Patterns of change in energy distribution with speed differed within the fin musculature as indicated by a significant speed-muscle type interaction (GLM, F_22,228_ = 1.78, p<0.05, *η* ^2^ = 0.15). A contrasts analysis, comparing estimated energy use at a given level to that at the adjacent lower speed detected changing energy costs in a number of fin muscles. Decreased energy costs were detected in parts of the pectoral, pelvic and dorsal fin musculature, while parts of the caudal fin musculature showed increased energy use (Fig. 5). Planned contrasts detected decreased absolute energy cost with increasing speed in parts of the musculature of the pectoral and pelvic girdles and dorsal fin, while estimated energy cost increased in parts of the caudal fin musculature (Fig. 5).

**Figure 6:**
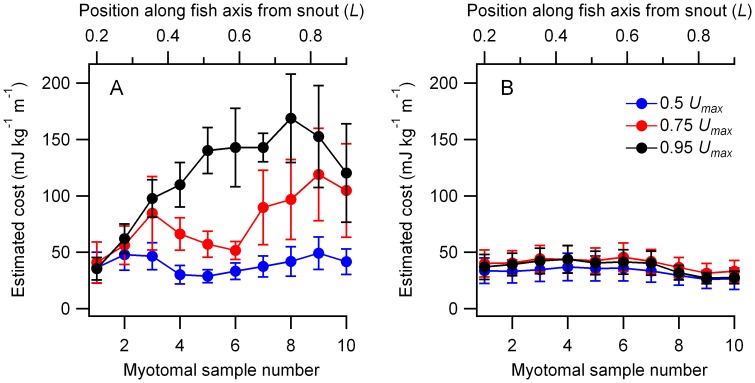
Estimated energy costs of activity within the myotomal musculature. A. Slow myotomal muscle. B. Mosaic myotomal muscle. Segmental energy estimates were obtained by multiplying the total estimated energy directed to a myotomal muscle type, expressed as a net cost of transport, by the proportion of the total blood flow received by an axial segment. Data are shown as mean ± sem (N = 6 to 8).

A segmental analysis of energy distribution was carried out for both the slow and mosaic myotomal muscle by dividing each muscle type into 10 axial segments. The energy directed to a muscle type within a segment was estimated by multiplying the total energy cost associated with that muscle type (Fig. 4B) by the proportion of its total blood flow received within that segment. We detected overall changes in energy costs with speed in the slow myotomal muscle (Fig. 6A, GLM, F_2,60_ = 42.30, p<0.001, *η* ^2^ = 0.59). We also detected an interaction between myotomal slow muscle position and speed (Fig. 6B, GLM, F_18,120_ = 1.87, p<0.05, *η* ^2^ = 0.22) indicating a change in the axial pattern of energy expenditure within the slow muscle with speed. No speed-position interaction was detected for the mosaic myotomal muscle (GLM, F_18,120_ = 0.09, p>0.05, *η* ^2^ = 0.01), and there was no detectable change in overall mosaic muscle energy expenditure (GLM, F_2,60_ = 3.48, p>0.05, *η* ^2^ = 0.06) with speed.

Relative distributions of blood flow to the viscera are shown in Table 1. The kidney and liver receive blood from both arterial and portal blood vessels. It was assumed that the microspheres found in these tissues were solely from arterial sources, but total blood flow to these organs may be greater than estimated. Microspheres found in the gills and heart reached them via the venous return. The low amounts found in these tissues confirm adequate capture of the injected microspheres in the systemic capillary beds.

**Table 1:** Relative distribution of microsphere estimated blood flow to the viscera.

	Percentage of total blood flow			
Organ	Rest	0.5 *U_max_*	0.75 *U_max_*	0.9 *U_max_*
Liver	2.96±1.61	1.24±0.38	0.60±0.33	0.16±0.04
Stomach	2.28±1.11	2.68±1.44	1.31±0.95	0.71±0.27
Intestine & pyloric caecae	9.77±3.80	6.25±1.83	5.31±2.45	2.93±1.12
Spleen	0.31±0.25	0.39±0.34	0.04±0.02	0.03±0.01
Swim Bladder	0.20±0.06	0.24±0.06	0.14±0.06	0.12±0.05
Kidneys	7.79±3.17	3.41±0.85	3.22±1.07	2.92±0.65
Gonads	1.12±0.41	1.43±0.98	0.65±0.37	0.24±0.12
Gills	0.76±0.19	0.73±0.21	0.44±0.13	0.38±0.12
Heart	0.14±0.06	0.13±0.07	0.08±0.04	0.07±0.03

Data are presented as mean ± sem (N = 6 to 8). Microspheres found in the gills and heart reached them via the venous return and do not reflect the total blood flow through these organs. The low amounts found in these tissues confirm adequate capture of the injected microspheres in the systemic capillary beds.

## Discussion

The high energetic costs of locomotion dominate the daily energy budgets of many animals. As such, locomotor energy costs and economy significantly influence organismal fitness. The majority of this cost is associated with muscle activity. However, the structural and functional complexity of locomotor systems limits understanding of the underlying factors that determine total energy expenditure. Although the transfer of muscle power output to increase the momentum of a fluid is the dominant mechanical factor in swimming, significant muscle masses are associated with roles other than power production. Without a clearer understanding of how total energy costs are allocated to particular mechanical functions, and how these may shift with changing speed or to counter the destabilizing effects of an unpredictable physical environment, the extent to which measures of total energy metabolism can be placed in context with behavior and environmental conditions in the field is limited. A means to identify the costs associated with activity in particular muscles or muscle groups is required in order to assign costs to specific mechanical functions. In the absence of this type of information, locomotor systems are essentially a black box from an energetic perspective. Rates of blood flow to skeletal muscle provide a proxy estimate of aerobic energy expenditure at the muscle level, allowing the total aerobic cost of swimming to be partitioned between different muscle types and their functional roles.

### Functional partitioning of overall swimming cost

The patterns of blood flow distribution to the skeletal musculature changed with swimming speed, both in terms of the proportion of cardiac output directed to different muscle types (Fig. 2), and how this was distributed within a particular category of skeletal muscle (Figs. 3, 5 & 6). As swimming speed increased from rest there was a progressive increase in the proportion of cardiac output directed to the slow myotomal muscle (Fig. 2). Proportional flows to the fin musculature increased from rest to the lowest swimming speed used, then declined (Fig. 2). These patterns can be used to allocate an approximate energetic cost within the context of total aerobic metabolism (Fig. 1) to activity within the myotomal and fin musculature.

Ideally, this requires initial identification of locomotor and non-locomotor costs within the total aerobic energy expenditure. This is a challenge common to all studies of net locomotor cost. The typical approach is to subtract a baseline value, usually basal or resting metabolism, or a multiple thereof, from the total metabolic rate during locomotion [15], [16], [22]. This is taken to represent the metabolic costs of ‘maintenance’ activities not directly associated with the activity of locomotor muscles. It is unlikely that non-muscle costs remain constant as the levels of muscular work and metabolism change with speed [22], [90], [91]. For example, an inability to maintain arterial blood pressure while simultaneously perfusing all vascular beds leads to a reduction in mass specific blood flow rates to non-muscle tissues during exercise, and redirection of cardiac output to muscle tissue. This is almost certainly associated with reduced non-locomotor energy expenditure, particularly at swimming speeds approaching *U_crit_* [74]. Unlike skeletal muscle tissue, the rate of blood flow to non-muscle tissue is generally a poor indicator of energy metabolism. For example, large increases in flow through the teleost splanchnic circulation occur after feeding, and although this supports energy metabolism in the gut it is also linked to increased transport of absorbed nutrients [92]. Similarly, kidney perfusion rates are linked to the control of glomerular filtration rate, not just the support of energy metabolism in kidney tissue [93]. This partial functional decoupling of blood flow rate from energy metabolism in the splanchnic organs and kidneys means that their blood flow rate cannot be used to estimate any change in non-locomotor cost. Given that the overall non-locomotor costs form an increasingly small proportion of total energy expenditure as speed increases, errors in net locomotor cost estimates associated with uncertainty surrounding the magnitude of the subtracted baseline are small, and we have therefore retained the practice of treating resting metabolic rate as an estimate of the appropriate value.

The estimated energy costs of supporting myotomal muscle function dominated net swimming costs across the experimental speed range (Fig. 4A, B). This is consistent with the role of the myotomal muscle as the primary power source during undulatory swimming [9], [44]. In most fish species a superficial zone of slow muscle fibers, recruited during steady, aerobic swimming is anatomically and functionally separated from deeper, fast muscle fibers that are only recruited during transient, high-speed maneuvers [17], [44], [47]. In salmonids, the functional division of the myotomes into distinct slow and fast muscle regions is not as clear-cut. The deeper myotomal muscle is a ‘mosaic’ consisting of fast muscle fibers interspersed with smaller diameter fibers that have some morphological and physiological similarities to slow muscle [48], [51], [52]. These may constitute approximately 5% of the myotomal muscle mass [49], an amount approximately equal to that of the superficial slow muscle fibers. If active during steady swimming, the interspersed mosaic fibers could potentially contribute to muscle power output.

Electromyograms have not provided clear information regarding the activity and function of salmonid mosaic muscle. In some cases, data are lacking, as activity was recorded solely from the superficial slow muscle with the implicit assumption that this was the primary power source for steady swimming [32], [94]. Where electrodes have been placed in the mosaic muscle during steady swimming, low intensity activity attributed to the small-diameter mosaic fibers has been described at low and intermediate speeds that is distinct from the intense activity associated with fast fiber recruitment at high speeds [55]. Hudson [54] also reports mosaic muscle activity at the relatively modest speed of 1 *L* s^−1^. Indirect evidence, such as fiber hypertrophy and increased mosaic muscle capillary density in response to sustained swimming, also suggest recruitment of mosaic muscle at intermediate speeds [51], [52], [53]. This contrasts however with data from Bone et al. [45] that show no mosaic activity until relatively high speeds around 2 *L* s^−1^.

Blood flow data show a relatively high proportion of total systemic blood flow being directed to the mosaic muscle (Fig. 2). At 0.5 *U_max_* proportional blood flows to the slow and mosaic muscle are equal. Mass specific flows are however much greater in the slow muscle given its much smaller total mass, and consistent with the higher capillary density in the slow compared to the mosaic muscle [53], [95]. In relative terms, flow to the mosaic muscle declines with swimming speed, but given the overall increase in cardiac output and energy expenditure with swimming speed, the estimated energy expenditure associated with mosaic muscle activity remains relatively stable (Figs. 4B & 6B). This could simply represent maintenance of resting metabolism in inactive muscle across the speed range, although changes in mass specific blood flow rates suggest otherwise. Absolute cardiac output was not measured directly in the present study, but flow cuff measurements from the ventral aorta in rainbow trout indicate a cardiac output range from 20 ml^−1^ min^−1^ kg^−1^ of body mass at rest, increasing to a maximum of approximately 75 ml^−1^ min^−1^ kg^−1^ [70], [96]. Based on our relative microsphere distributions and mosaic muscle masses, and assuming a similar increase in cardiac output from rest to 0.95 *U_max_* in the present study, this would yield mass specific blood flow estimates of approximately 1.1 ml^−1^ min^−1^ 100 g^−1^ of mosaic muscle tissue at rest and 2.0 ml^−1^ min^−1^ 100 g^−1^ at 0.95 *U_max_*. A substantial increase relative to rest, and similar to the absolute values and factorial increases obtained for trout mosaic muscle using concurrent microsphere and flow cuff cardiac output measurements [55], [70]. This apparent increase in mosaic muscle mass-specific blood flow and energy expenditure from rest to swimming, without a further change with speeds beyond 0.5 *U_max_* is consistent with low levels of activity within the mosaic muscle fibers. However, given their dispersal throughout the myotomes and the proximity of deeper fibers to the vertebral column it is unlikely that they can contribute appreciably to body bending and power transfer during steady swimming.

A regional analysis of blood flow and energy expenditure in both the slow and mosaic myotomal muscle was made by dividing the myotomal muscle into 10 axial segments (Fig. 6). During swimming, the pattern of energy delivery to the slow muscle shifted. At lower speeds a relatively high proportion of the slow myotomal energy costs were associated with the anterior myotomes (Fig. 6A). As speed increased further, the additional energy required was primarily directed to the posterior slow muscle (Fig. 4). The anterior to posterior shift in energy delivery may be associated with two factors: changes in the mechanical power requirements of swimming, and a changing role of the myotomal muscle in stabilizing swimming trajectory. The distribution of slow myotomal muscle in trout is skewed towards the posterior half of the body axis, with its greatest cross sectional area coinciding with approximately 0.7 *L* from the snout [44]. The mass specific power output of the slow muscle also increases moving from anterior to posterior myotomes [17]. This is due to an increased work output per tail beat cycle associated with greater muscle strains in the posterior muscle [6], [17]. Increased energy expenditure in the posterior myotomes is therefore consistent with axial differences in the capacity of the myotomal muscle to deliver the increased mechanical power requirements of higher speed swimming. Swimming at low speeds likely requires greater active stabilization of the swimming trajectory as trimming forces created by the fins are relatively low [40], [41]. The anterior myotomes may play a role in this during lower speed undulatory swimming (Fig. 6A). In cod, the anterior myotomal muscle shows a high degree of variation in activity across tail beat cycles relative to the posterior muscle [42]. This is not consistent with the role of a motor supplying steady swimming power, but more likely associated with making small corrections to body posture to stabilize the heading of the fish [42]. Our estimates of axial energy distribution therefore suggest a regional differentiation in the mechanical function of slow myotomal muscle, with the functional and energetic emphasis shifting from anterior stabilization to increased posterior power production as swimming speed increases. In contrast, the mosaic myotomal muscle shows no detectable change in energy expenditure with speed, further suggesting a modest mechanical contribution during steady swimming.

The relative and absolute proportion of estimated swimming cost associated with fin muscle activity initially declined and then plateaued with increasing swimming speed (Fig. 4A, B). At the lowest swimming speed these costs were substantial, approaching 20% of the net cost of swimming. At high speeds fin muscle costs became relatively trivial in relation to the energy expenditure associated with the myotomal muscle, representing only 5% of the total (Fig. 4A) and an approximate halving of the absolute fin muscle energy use relative to 0.5 *U_max_* (Fig. 4B). Median and paired fins potentially serve a number of functions during undulatory swimming. The caudal fin is largely associated with the transfer of mechanical power to the wake. Other median fins may also contribute to thrust production through the interaction of their wakes with that generated by body and caudal fin undulation [34], [97]. As with the myotomal muscle segments, the relative functional importance of a particular fin in steady swimming should be reflected by a shift in the cost of activity associated with the fin. The estimated total cost of fin muscle activity (Fig. 4B) was combined with measured blood flows to individual fin muscles or muscle groups (Fig. 3) to provide an estimate energy distribution within the fin musculature (Fig. 5).

Relative blood flows to the pectoral and pelvic fin musculature remained relatively static with increasing speed (Fig. 3). Combined with the overall decline in fin muscle energy expenditure this resulted in reduced estimated energy use with increasing speed (Fig. 5). This is consistent with their observed kinematics during steady swimming, where the fins are largely abducted against the body, primarily being employed during transient, unsteady maneuvers or at very low speeds [33], [36], [37]. Similar decreases in estimated energy use were seen in the dorsal fin musculature, particularly the protractor and interradialis dorsalis (Fig. 5). At low swimming speeds trout erect and may actively modulate the shape and stiffness of the dorsal fin in synchrony with the tail beat cycle. In doing so the fin sheds a wake that interacts with that of the caudal fin to potentially enhance thrust production [34]. At higher speeds the fin is retracted and does not contribute significantly to the wake. Our energy estimates are also indicative of a shift in dorsal fin function with speed. At low speeds there is an energy cost associated with the role of the dorsal fin as an auxiliary thrust producer, but energy usage by the fin muscles decline at higher speeds as it no longer contributes to swimming in this way. The only fin muscles that showed an increase in relative blood flow and estimated energy use with speed were those associated with the caudal fin (Fig. 5). The shape, and likely the stiffness of the caudal fin is actively modulated throughout the tail beat cycle during steady swimming [35]. Increased requirements for power transfer to the wake with increasing speed should impose a greater hydrodynamic load on the fin, requiring increased muscle activity to stiffen the fin rays. Our data indicate the energetic and mechanical importance of the caudal fin as an actively modulated propulsor, not just a passive element for power transmission from the musculature to the wake.

The estimates of energy cost to support fin muscle activity are likely to represent minimal values in comparison to field conditions. Given the opportunity to select steady swimming speeds in flowing water, brook trout swim at less than 1 *L* s^−1^ [98]. Similarly low average speeds are maintained during routine swimming through still water [99]. Low speeds are associated with increased requirements for active control of trajectory and posture [36], [37], [40], [41]. Given these mechanical constraints and the decreased energy cost of fin muscle activity indicated by blood flow rates (Fig. 4), the costs of fin muscle activity are likely to become increasingly high at speeds below those used in the present study. In addition, volitional swimming behaviors are rarely steady. Accelerations, decelerations and turning maneuvers dominate the repertoire of swimming behaviors [98], all of which require active modulation of fin position, area and stiffness. Finally, unsteady and turbulent flows in the field are likely to further increase energy use by fin muscles. Vorticity destabilizes fish, potentially requiring corrective action through fin stabilization [100], [101]. These demands may increase the energy costs of swimming and reduce maximum swimming performance [102], [103]. Our data therefore represent a benchmark against which changes in cost associated with unsteady swimming conditions can potentially be compared, with changes in muscle blood flow and energy use from steady to unsteady flow regimes highlighting the mechanical factors associated with altered energy use.
